# An Explainable Machine Learning Model for Material Backorder Prediction in Inventory Management

**DOI:** 10.3390/s21237926

**Published:** 2021-11-27

**Authors:** Charis Ntakolia, Christos Kokkotis, Patrik Karlsson, Serafeim Moustakidis

**Affiliations:** 1 Machining Technology and Production Management, Sector of Materials Engineering, Department of Aeronautical Studies, Hellenic Air Force Academy, 13672 Tatoi, Greece; 2 School of Naval Architecture and Marine Engineering, National Technical University of Athens, 15772 Athens, Greece; 3 TEFAA, Department of Physical Education and Sport Science, University of Thessaly, 42100 Trikala, Greece; chkokkotis@gmail.com; 4 AIDEAS OÜ, Narva mnt 5, 10117 Tallinn, Estonia; p.karlsson@aideas.eu (P.K.); s.moustakidis@aideas.eu (S.M.)

**Keywords:** prediction models, post-hoc explainability, inventory backorder prediction, inventory management

## Abstract

Global competition among businesses imposes a more effective and low-cost supply chain allowing firms to provide products at a desired quality, quantity, and time, with lower production costs. The latter include holding cost, ordering cost, and backorder cost. Backorder occurs when a product is temporarily unavailable or out of stock and the customer places an order for future production and shipment. Therefore, stock unavailability and prolonged delays in product delivery will lead to additional production costs and unsatisfied customers, respectively. Thus, it is of high importance to develop models that will effectively predict the backorder rate in an inventory system with the aim of improving the effectiveness of the supply chain and, consequentially, the performance of the company. However, traditional approaches in the literature are based on stochastic approximation, without incorporating information from historical data. To this end, machine learning models should be employed for extracting knowledge of large historical data to develop predictive models. Therefore, to cover this need, in this study, the backorder prediction problem was addressed. Specifically, various machine learning models were compared for solving the binary classification problem of backorder prediction, followed by model calibration and a post-hoc explainability based on the SHAP model to identify and interpret the most important features that contribute to material backorder. The results showed that the RF, XGB, LGBM, and BB models reached an AUC score of 0.95, while the best-performing model was the LGBM model after calibration with the Isotonic Regression method. The explainability analysis showed that the inventory stock of a product, the volume of products that can be delivered, the imminent demand (sales), and the accurate prediction of the future demand can significantly contribute to the correct prediction of backorders.

## 1. Introduction

Backorder occurs when a product is temporarily unavailable or out of stock and the customer places an order for future production and shipment [1]. Backorders are noticed mainly in case of product unavailability due to excessive demand or future release on the market. For instance, COVID-19 and lockdown measures raised the need for antiseptic products and indoor domestic activities that led to a mass wave of online purchases. This trend led to the bullwhip effect, or else the Forrester effect, for many industries and companies that had not succeeded on predicting the increase demand. The stock of products proved insufficient; however, due to products’ low availability and alternative solutions, the customers were willing to wait for their order. Another recent example is the early announcement of the upcoming release in the market of a new product from a famous company. In that case, the company accepts backorders from customers, since the initial production quantity will be insufficient to cover the expected demand for the popular product [1]. The ability of a company to address backorders impacts significantly the company’s revenue, share market price, and customers’ trust [2].

The backorders of products play a crucial role in the management of the inventory, since they affect the total production costs of the whole supply chain. In the literature, various studies addressing the Economic Order Quantity (EOQ) and Economic Production Quantity (EPQ) have been published, taking into account backorders [3,4,5,6]. These approaches include: (i) the coordination and minimization of total costs of the supply chain with backorders [7,8] among other factors, such as with stochastic supply distribution [9,10]; (ii) the inventory problem addressed with backorders [11,12,13,14] and safety stocks [15], multi-objective optimization formulations with fixed backorder, and time-weighted backorder [16], stochastic demand and price discount [17,18], the integration of human errors [19], customers’ preferences [20], or customers’ behavior [21], and from the energy-efficient perspective with the aim to minimize carbon emissions [22]; (iii) fuzzy logic to model the demand or the order quantity for finding the optimal stock quantity [23,24,25]; (iv) heuristic approaches for optimizing the inventory systems [26,27,28].

Due to the importance of backorders and their impact on the whole supply chain costs, studies have been focused on the prediction of inventory backorders. To address the issue of backorders prediction, artificial intelligent techniques have been employed to deal with imbalanced data issues, since the number of products going on backorder is much lower than that of those that are on stock [29]. A machine learning approach was proposed [30] to maximize the expected profit of backorder decisions by integrating the profit-based measure into the prediction model and optimizing the decision threshold. In this context, various machine learning models were evaluated, such as Logistic Regression (LR) and k-Nearest Neighbor (KNN) classifiers, Decision Tree, Support Vector Machine (SVM), and Multi-Layer Neural Network (NN). Another machine learning approach based on Distributed Random Forest and Gradient Boosting Machine learning techniques was presented for predicting the probable backorder scenarios in the supply chain [2]. Unsupervised learning was used to predict backorders by using Deep Autoencoder [29]. Deep neural networks for imbalanced data were proposed for backorder prediction [1]. A case study on the Danish Craft Beer Breweries was presented by using machine learning models for predicting the backorders [31].

The above studies employed machine learning methods to address the backorder prediction problem, whether a product would be backordered or not. However, none of these aimed to explain and interpret the impact of features on the prediction output. To this end, this study focused on developing an explainable machine learning pipeline for: (i) evaluating the performance of well-known machine learning models to predict backorders as a binary classification problem and (ii) interpreting the results by using a post-hoc explainability model (SHAP) on the best performing model. Special notice was given to the treatment of the imbalanced dataset by using an undersampling technique.

## 2. Materials and Methods

### 2.1. Dataset

In this study, the publicly available dataset ‘Predict Product Backorders. Can you predict product back orders?’ (https://www.kaggle.com/c/untadta/data (accessed on 1 October 2021)), that was initially created for a competition, was used. In total, 23 features are included in the dataset. Out of the 23 features given in the dataset (Table 1), 15 are numerical, and 8 (including the target variable “went on back order”) are categorical features. The data consisted of 9714 products that were backordered and 1,038,860 that were not. 

### 2.2. Mehtodology

**Figure 1:**
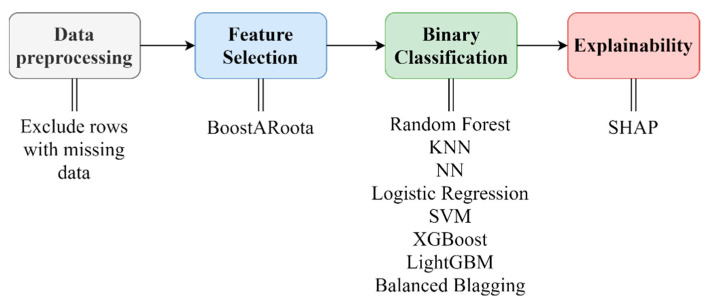
Methodology steps.

The presented dataset was used in a machine learning (ML) pipeline to predict possible backorders in the inventory management system. The steps integrated in the ML pipeline were the following (Figure 1): (i) data preprocessing to handle the missing data and the categorical values; (ii) feature selection via a state-of-the-art method, called BoostARoota [32,33]; (iii) a comparative evaluation of popular machine learning models, such as Random Forest (RF), LightGBM (LGBM), XGBoost (XGB), Balanced Blagging (BB), Neural Networks (NN), Logistic Regression (LR), Support Vector Machines (SVM), and K-Nearest Neighbors (KNN); (iv) an explainability analysis with the use of the SHAP model applied to the best-performing prediction model in (iii).

For the preprocessing of the dataset, we deleted the rows with missing values, so that we had the maximum possible real information. In addition, we normalized the data set to [0,1]. Finally, to address the problem of imbalanced data, we reduced the samples of the majority class to reach the number of samples in the minority class.

Regarding the feature selection process, the state-of-the-art selection method BoostARoota was used. It is a fast XGBoost wrapper feature selection algorithm that follows the Recursive Feature Elimination approach. It operates similarly to Boruta utilizing XGBoost as the base model. BoostARoota returns an optimal subset of features by eliminating up to 10% of the initial set of features. Its effectiveness has been proven in various applications [32,33]. A 10-fold cross validation was performed for the selection of the most important features.

The comparative evaluation included 8 popular and commonly used classifiers, such as Random Forest (RF) [34], K-Nearest Neighbor (KNN) [35], Neural Networks (NN) [36], Logistic Regression (LR) [37], Balanced Blagging (BB) [38], Support Vector Machines (SVM) [39], XGBoost [40], and LightGBM [41]. A 70/30% validation strategy was employed to generate the training and testing sets with an integrated cross validation strategy that employed grid search for the hyperparameter tunning to avoid overfitting and bias error. In Table 2, a description of the employed hyperparameters is presented. For the performance evaluation of the models, the accuracy, recall, f1-score, precision, AUC metrics were used.

Following the results from the validation of the models, the classifiers with similar performance were calibrated to increase their performance and identify the best one. Calibration is a post-processing operation, which improves the probability estimation of a model [42,43]. To calibrate the models, the Platt Scaling (sigmoid) [44] and Isotonic Regression [45,46] (isotonic) methods were adopted.

A post-hoc explainability was finally applied on the best performing model based on the SHapley Additive exPlanations (SHAP) model to explain the predictive model and the contribution of the most important features. SHAP is a game theory approach typically employed to explain the output of any machine learning model. It connects optimal credit allocation with local explanations using the classic Shapley values from game theory and their related extensions [47,48,49].

## 3. Results

In this section, the results of each step of the ML methodology are presented.

### 3.1. Feature Selection

The BoostARoota algorithm selected the following features as important in random order of appearance:

- national inv
- lead time
- in transit qty
- forecast_3_month
- forecast_6_month
- forecast_9_month
- sales_1_month
- sales_3_month
- sales_6_month
- sales_9_month
- min bank
- pieces past due
- perf_6_month_avg
- perf_12_month_avg
- local bo qty
- deck risk
- ppap risk

### 3.2. Classification

#### 3.2.1. Validation

**Figure 2:**
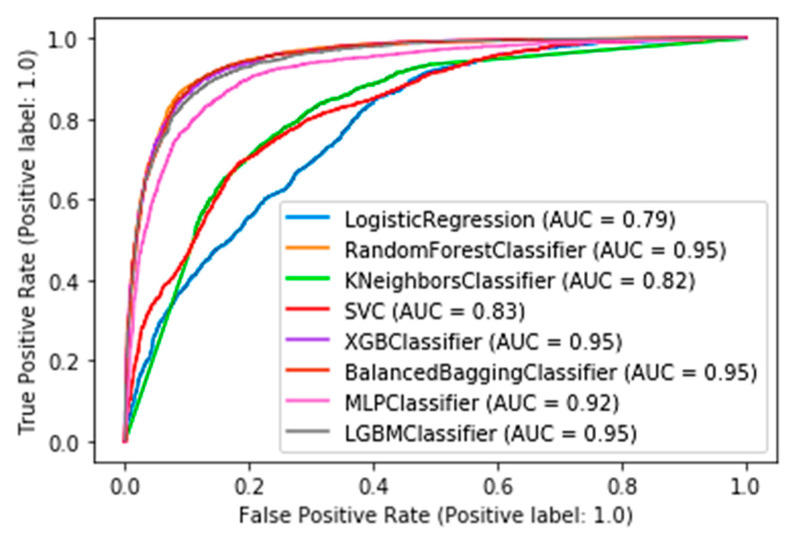
Roc curves of the competitive ML models.

In this subsection, the results from the comparative evaluation of the ML models are presented. Table 3 shows the best metric scores of each classifier used in this study with their hyperparameters tuning. Furthermore, the roc curves and AUC scores are presented in Figure 2. 

#### 3.2.2. Calibration

**Figure 3:**
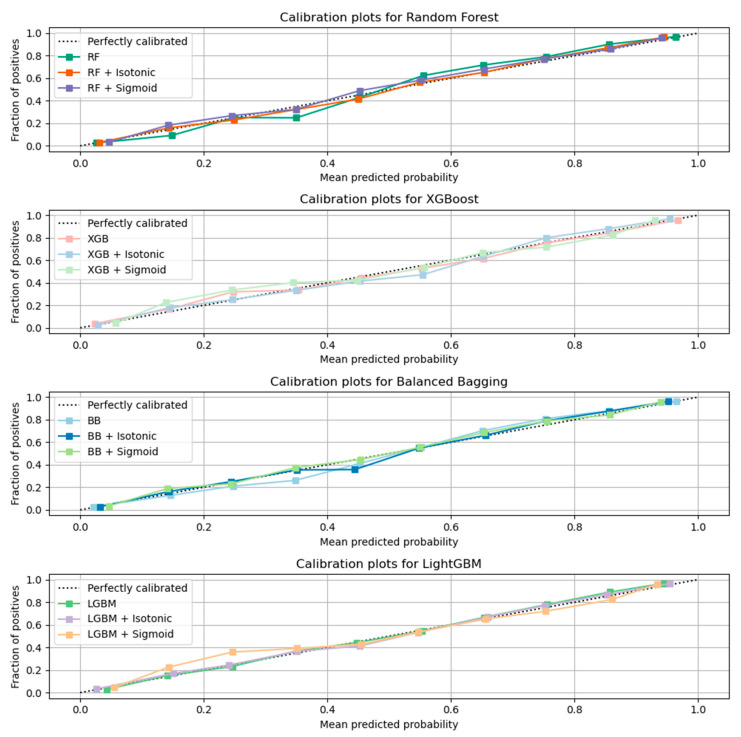
Calibration results for each classifier with comparative performance based on AUC results.

**Figure 4:**
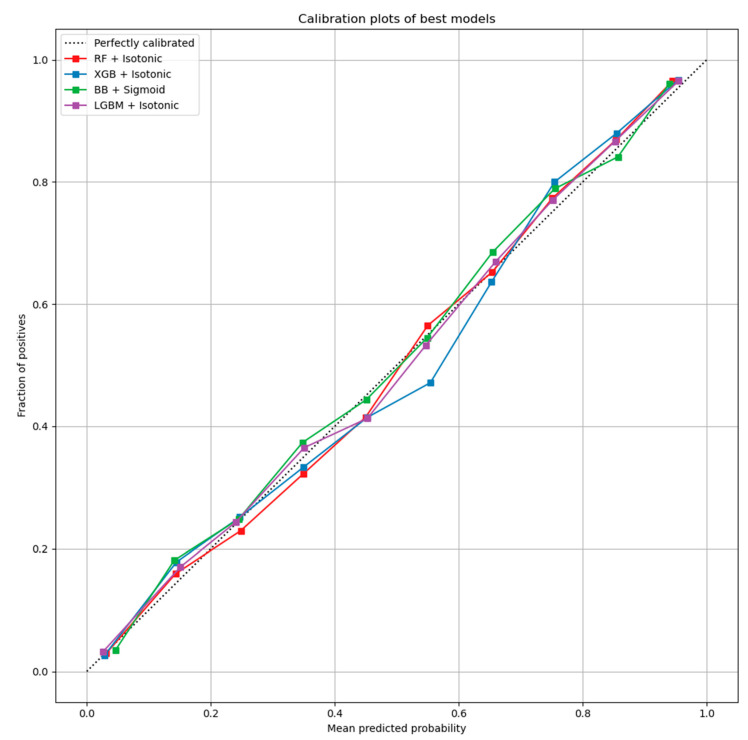
Best calibrated models for each classifier.

**Figure 5:**
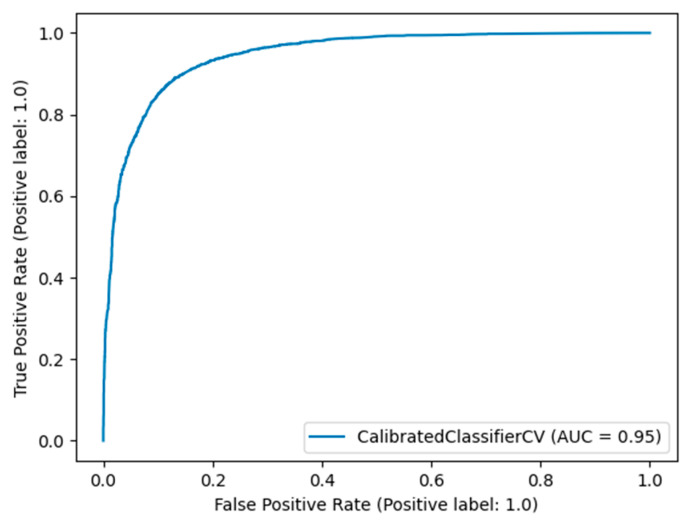
Roc curve of the LGBM + Isotonic model.

Here, the results from the calibration process are shown. Figure 3 depicts the calibration plots for each of the best performing models that achieved similar performance (RF, LGBM, XGB, and BB). In each plot, the perfectly calibrated line (dot line), the initial model, and the model calibrated with the Platt’s method (sigmoid) and the Isotonic Regression (isotonic) method are presented. For each model, the best calibrated one that best fitted the dot line was then used for a comparison, illustrated in Figure 4. Figure 5 shows the Roc curve of the best overall calibrated model (LGBM + Isotonic).

### 3.3. Explainability

**Figure 6:**
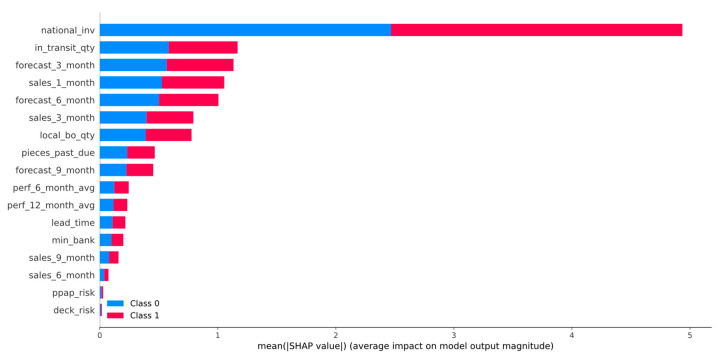
SHAP summary plot of LightGBM calibrated with the Isotonic Regression method.

**Figure 7:**
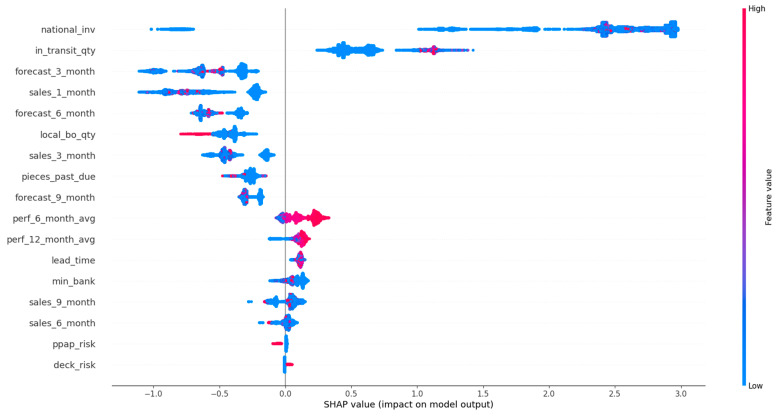
Beeswarm plot of LightGBM calibrated with the Isotonic Regression method for the backordered class.

**Figure 8:**
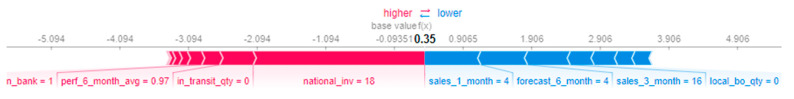
Product correctly classified as non-backordered.

**Figure 9:**
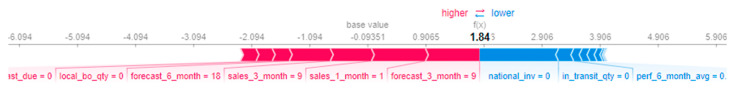
Product correctly classified as backordered.

In this section, the results of the SHAP analysis are presented. Figure 6 illustrates the summary plot of LGBM calibrated with the Isotonic Regression method, while in Figure 7, the beeswarm plot for the backordered class is shown. Furthermore, Figure 8 and Figure 9 show two examples for products that were classified correctly as backordered and non-backordered, respectively.

## 4. Discussion

The BoostARoota feature selection method selected 17 out of 23 features that formed the initial dataset. These features were used to form the final dataset for training, validation, and testing of the proposed ML pipeline in this study. They were relevant to inventory stock, transit information, sales forecast, and sales quantity (Section 3.1).

Eight machine learning models were used for a comparative evaluation (Table 3). Among these models RF, XGB, LGBM, and BB presented similar performance based on the AUC score (0.95, Figure 2). Specifically, Table 3 summarizes the metric scores, the confusion matrixes, and the selected hyperparameters of the employed ML models for this binary problem. The majority of the employed classifiers achieved accuracy up to 88.85% in comparison with KNN, LR, and SVM which achieved lower accuracy (up to 75.93%). The RF, XGB, LGBM, and BB models also achieved high performances in the remaining metrics such as recall (up to 90.69%), f1-score (up to 89.12%), and precision (up to 88.10%) scores. From the confusion matrixes of the aforementioned ML models, it turned out that the ML models work satisfactorily in this task.

The RF, XGB, LGBM, and BB models that achieved comparative performance were calibrated based on isotonic and sigmoid methods. Figure 3 illustrates the initial models and their calibration with the aforementioned calibration methods. The results showed that for RF, XGB, and LGBM, the calibration with the Isotonic Regression method reached better results, while for the BB classifier, the Platt Scaling (Sigmoid) method presented better performance. From the comparative evaluation, depicted in Figure 4, the LGBM classifier calibrated with the Isotonic Regression method presented the best overall performance, as it is asymptotically closer to the dotted line that represents the perfectly calibrated model (Figure 4 and Figure 5). 

Figure 6 presents the features’ impact on the output of the best model (LightGBM + Isotonic) for the proposed dataset. The features were sorted by the mean absolute value of the SHAP values which represent the SHAP global feature importance. Furthermore, the most important features that significantly affected the prediction output of the model were the national_inv, the in_transit_qty, the forecast_3_month, the sales_1_month, and the forecast_6_month. The national inv concerns the current inventory level of components. The feature in transit qty describes the quantity in transit, and the sales_1_month concerns the sales quantity in the prior month. The features forecast_3_month and forecast_6_month relate to the forecast sales for the next 3 and 6 months. 

Figure 8 shows that the topmost influential features n_bank, perf_6_month_avg, in transit, national_inv, sales_1_month, forecast_6_month, sales_3_month, and loval_bo_qty led to the prediction value of 0.35, which was transformed to 1. The features that are indicated with red color influenced positively, which means that they dragged the value closer to 1, while the features in blue color had the opposite effect. Similarly, for an example of a backordered product, Figure 9 shows the values of the top influential features that pushed the product to the backordered class. It is observed that lower values of inventory stock, products’ quantity received, and source performance of the last 6 months and higher values of forecasts and sales pushed the output prediction to the non-backordered class.

To interpret the results from a managerial perspective based on the beeswarm plot illustrated in Figure 7, a product with low stock and high short-term and mid-term future demand will probably be backordered, since the inventory stock will not be able to satisfy the customers’ demand, and at the same time, the expected quantity of products to be delivered to the inventory is also low (Figure 7). Therefore, it is shown that an optimal management of an inventory system that can handle and prevent the forthcoming backorders of products incorporates: (i) accurate predictions on future demands of products, so appropriate decisions can be made on the inventory stock of the product and on product production on time; (ii) increase of the products’ quantity in transit and/or decrease of the transit time by re-scheduling on time the transportation planning and logistics; (iii) the product performance, which means that if the product’s quality satisfies the customers’ requirements, the demand of this product is expected to be increased.

## 5. Conclusions

Businesses target to increase their profit by retaining low production costs trying in parallel to provide quality service for customer satisfaction. An important part of the production costs is related to the inventory management system. Therefore, it is of high importance to effectively and accurately predict various issues that could occur, leading to additional costs and causing a negative impact on the inventory management system and business operation. One of these issues is product backorder. When a product is backordered, the production should be rescheduled in order to address the demand. This adds additional costs to the business operation. To deal with the backorder issue, this study considered two key aspects: (i) the development of an accurate prediction model for product backorder via a comparative evaluation of popular classifiers and model calibration, and (ii) a post-hoc analysis to explain and interpret the major contributing factors that lead to product backorder.

Specifically, this study tackled the problem of predicting products that will be backordered in an inventory management system. This problem is usually evaluated as a highly imbalanced binary classification problem. Due to the large volume of data, an under-sampling approach was initially adopted to solve this issue. A machine learning pipeline, based on a comparative evaluation of eight popular classifiers, was then adopted, followed by a calibration process applied to the models with similar performance and an explainability analysis of the best-performing model. The results showed that four models achieved almost comparable performance based on AUC scores and other metrics (Table 3). Specifically, the RF, XGB, LGBM, and BB models reached an AUC score of 0.95 (Figure 2). These models were calibrated with the Platt’s and Isotonic Regression methods. The LGBM model calibrated with the Isotonic Regression method presented a slightly better calibration for our data (Figure 4). For this model, post hoc explainability based on the SHAP model showed that the features most contributing to the prediction output of the model relevant to the current inventory level of the component were the quantity in transit and the short-term and mid-term sales quantity and forecast sales (Figure 6). Backorders impact the costs that are linked to production, since the production schedule should be altered in order to deal with the demand of backordered products. Therefore, from the above analysis, it is shown that the decisions that will be made regarding the inventory stock of a product can significantly contribute to the optimal operation of an inventory management system. This decision should be made based on the volume of products that can be delivered, the imminent demand (sales), and the accurate prediction of the future demand.

A limitation of this study is the use of resampling techniques to cope with imbalanced data. To this end, future work will include the use of Siamese neural networks that have proven effective in case of imbalanced datasets.

## Figures and Tables

**Table 1:** Dataset description.

Feature Name	Description	Type
national inv	Current inventory level of component	Numerical
lead time	Transit time	Numerical
in transit qty	Quantity in transit	Numerical
forecast x month	Forecast sales for the net 3, 6, 9 months, where x represents the months	Numerical
sales x month	Sales quantity for the prior 1, 3, 6, 9 months, where x represents the months	Numerical
min bank	Minimum recommended amount in stock	Numerical
potential issue	Indictor variable noting potential issue with item	Categorical
pieces past due	Parts overdue from source	Numerical
perf x months avg	Source performance in the last 6 and 12 months, where x represents the months	Categorical
local bo qty	Amount of stock orders overdue	Numerical
X17–X22	General Risk Flags	
deck risk, oe constraint, ppap risk, stop auto buy, rev stop	Different Flags (Yes or No) set for the product	Categorical
went on back order	Product went on backorder	Categorical

**Table 2:** Hyperparameters of the selected ML models.

Classifiers	Hyperparameters
RF	criterion: [gini, entropy], n estimators: [10, 15, 20, 25, 27, 30], min samples leaf: [1, 2, 3, 4, 5], min samples split: [2, 3, 4, 5, 6, 7]
KNN	n neighbors: [3, 4, 5, 7, 9, 12, 14, 15, 16, 17], leaf size: [1, 2, 3, 5], weights: [uniform, distance], algorithm: [auto, ball tree, kd tree, brute]
NN	hidden layer sizes: [(2, 5, 10), (5, 10, 20), (10, 20, 50)], activation: [tanh, relu], solve: [sgd, adam], alpha: [0.0001, 0.05], learning rate: [constant, adaptive]
RL	penalty = [11, 12], C: [0, 1, 2, 4, 6, 8, 10]
SVM	C: [0.001, 0.01, 0.1, 1, 2, 3, 4, 5, 6, 7, 8, 9, 10, 11, 12, 13, 14, 15], kernel: [linear,sigmoid,rbf,poly]
XGB	max depth: [1, 2, 3, 4, 5, 6, 7, 8, 9, 10], min child weight: [1, 2, 3, 4, 5, 6, 8, 10], gamma: [0, 0.4, 0.5, 0.6, 0.7, 0.8, 0.9, 1]
LGBM	n estimators: range (200, 600, 80), num leaves: range (20, 60, 10)
BB	n estimators: [10, 50, 100, 300, 500, 1000, 1100, 1200, 1300, 1400, 1500]

**Table 3:** Best metric scores of each ML model and the selected hyperparameters of each model.

Classifiers	Accuracy (%)	Recall (%)	F1-Score (%)	Precision (%)	Confusion Matrix			Hyperparameters
RF	88.82	89.94	89.01	88.10		0	1	Criterion = entropy, min samples leaf = 1, min samples split = 5, n estimators = 30
					0	2432	342	
					1	283	2531	
KNN	75.93	79.82	76.96	74.30		0	1	algorithm = auto, leaf size = 1, n neighbors = 3, weights = distance
					0	1997	777	
					1	568	2246	
NN (MLP)	85.68	85.54	85.75	85.96		0	1	activation = tanh, alpha = 0.0001, hidden layer sizes = (10, 20, 50), learning rate = constant, solver = adam
					0	2381	393	
					1	407	2407	
LR	70.22	74.09	71.48	69.04		0	1	penalty = l2, C = 10.0
					0	1839	935	
					1	729	2085	
SVM	72.39	85.86	75.80	67.85		0	1	C = 15, kernel = rbf
					0	1629	1145	
					1	398	2416	
XGBoost	88.53	90.26	88.80	87.38		0	1	gamma = 0.7, max depth = 9, min child weight = 1
					0	2407	367	
					1	274	2540	
LightGBM	87.78	89.02	88.00	87.01		0	1	n estimators = 520, num leaves = 50
					0	2400	374	
					1	309	2505	
BB	88.85	90.69	89.12	87.61		0	1	n estimators = 1100
					0	2413	361	
					1	262	2552	

## Data Availability

The dataset used in this study can be found at https://www.kaggle.com/c/untadta/data (accessed on 23 November 2021).
